# Permafrost Dynamics Observatory—Part I: Postprocessing and Calibration Methods of UAVSAR L‐Band InSAR Data for Seasonal Subsidence Estimation

**DOI:** 10.1029/2020EA001630

**Published:** 2021-07-27

**Authors:** Roger J. Michaelides, Richard H. Chen, Yuhuan Zhao, Kevin Schaefer, Andrew D. Parsekian, Taylor Sullivan, Mahta Moghaddam, Howard A. Zebker, Lin Liu, Xingyu Xu, Jingyi Chen

**Affiliations:** ^1^ Department of Geophysics Colorado School of Mines Golden CO USA; ^2^ Jet Propulsion Laboratory California Institute of Technology Pasadena CA USA; ^3^ Viterbi School of Engineering University of Southern California Los Angeles CA USA; ^4^ National Snow and Ice Data Center Cooperative Institute for Research in Environmental Sciences University of Colorado at Boulder Boulder CO USA; ^5^ Department of Geology and Geophysics University of Wyoming Laramie WY USA; ^6^ Department of Civil & Architectural Engineering University of Wyoming Laramie WY USA; ^7^ Department of Geophysics Stanford University Stanford CA USA; ^8^ Earth System Science Programme Faculty of Science The Chinese University of Hong Kong Hong Kong China; ^9^ Department of Aerospace Engineering and Engineering Mechanics University of Texas Austin TX USA

**Keywords:** InSAR, UAVSAR, synthetic aperture radar, permafrost, active layer thickness, Arctic and boreal

## Abstract

Interferometric synthetic aperture radar (InSAR) has been used to quantify a range of surface and near surface physical properties in permafrost landscapes. Most previous InSAR studies have utilized spaceborne InSAR platforms, but InSAR datasets over permafrost landscapes collected from airborne platforms have been steadily growing in recent years. Most existing algorithms dedicated toward retrieval of permafrost physical properties were originally developed for spaceborne InSAR platforms. In this study, which is the first in a two part series, we introduce a series of calibration techniques developed to apply a novel joint retrieval algorithm for permafrost active layer thickness retrieval to an airborne InSAR dataset acquired in 2017 by NASA's Uninhabited Aerial Vehicle Synthetic Aperture Radar over Alaska and Western Canada. We demonstrate how InSAR measurement uncertainties are mitigated by these calibration methods and quantify remaining measurement uncertainties with a novel method of modeling interferometric phase uncertainty using a Gaussian mixture model. Finally, we discuss the impact of native SAR resolution on InSAR measurements, the limitation of using few interferograms per retrieval, and the implications of our findings for cross‐comparison of airborne and spaceborne InSAR datasets acquired over Arctic regions underlain by permafrost.

## Key Points

- We develop and present several calibration and postprocessing methods for seasonal subsidence estimation from interferometric synthetic aperture radar deformation
- Novel methods for phase referencing and uncertainty quantification due to nonergodicity within the multilook window are proposed
- Residual sources of uncertainty in active layer thickness estimation are discussed and quantified

## 1 Introduction

Air temperatures in the Arctic high‐latitudes are increasing at nearly twice the global rate, making the Arctic the fastest‐changing region in the global climate system (Jorgenson et al., 2001). Permafrost, or perennially frozen ground, covers approximately 24% of the continental land area within the Northern hemisphere (Zhang et al., 2000). Permafrost regions are one of the globe's largest reservoirs of carbon, containing 60% of the world's soil‐bound carbon (Hugelius et al., 2014; Turetsky et al., 2019). Sustained, interannual warming can induce significant changes to the seasonal freezing and thawing of the active layer—the surface soil layer immediately overlying the permafrost table. Due to both their disproportionate importance in the global carbon and climate cycles and their status as the fastest‐warming region on Earth, there exists a critical need for large‐scale monitoring efforts and vulnerability assessments of boreal and Arctic environments throughout the Northern hemisphere.

Interferometric synthetic aperture radar (InSAR) is a geodetic technique capable of resolving centimetric‐scale deformation of the surface of the Earth (Goldstein & Zebker, 1987; Rosen et al., 2000) with millimetric precision when sufficiently large interferogram networks are available (Berardino et al., 2002). During seasonal thaw of the active layer, the change in volume experienced by porebound water as it undergoes a phase change from solid to liquid results in subsidence of the ground surface, followed by uplift as the active layer refreezes in autumn (Chen et al., 2020; Liu et al., 2010). Liu et al. (2012) and Schaefer et al. (2015) developed the remotely sensed active layer thickness (ReSALT) algorithm, which utilized spaceborne InSAR measurements of the seasonal deformation of the thawing active layer to estimate the total active layer thickness. Similar studies have employed InSAR to quantify seasonal subsidence in periglacial environments (Antonova et al., 2018; Bartsch et al., 2019; Chen, Günther, et al., 2018; Chen, Liu, et al., 2018; Strozzi et al., 2018), long‐term deformation trends associated with thermokarst (Daout et al., 2020; Eshqi Molan et al., 2018; Iwahana et al., 2016; Liu et al., 2014), post‐fire evolution of the active layer (Michaelides, Schaefer, et al., 2019), motion of debris‐covered glaciers (Rouyet et al., 2019), thaw slumps and slope mass‐wasting (Bernhard et al., 2020; Dini et al., 2019), and active layer thickness water storage dynamics (Chen et al., 2020; Wang & Schmugge, 1980).

In 2015, NASA's Terrestrial Ecology Program initiated the Arctic‐Boreal Vulnerability Experiment (ABoVE), a major field campaign dedicated to studying large‐scale environmental change across Alaska and Western Canada. As part of the ABoVE campaign, NASA conducted a series of airborne remote sensing campaigns. During the 2017 airborne campaign, NASA collected a series of L‐band uninhabited aerial vehicle synthetic aperture radar (UAVSAR) InSAR data and P‐band Airborne Microwave Observatory of Subcanopy and Subsurface polarimetric synthetic aperture radar (PolSAR) data over much of the ABoVE study domain (Miller et al., 2019). The 2017 flight campaign consisted of measurements collected in spring between April and June, and autumn between September and November.

The Permafrost Dynamics Observatory (PDO) project combines the L‐band and P‐band data to simultaneously measure active layer thickness (ALT) and vertical profiles of volumetric water content. The PDO algorithm uses L‐band InSAR to measure seasonal surface subsidence due to the thawing of the active layer and the P‐band backscatter to measure soil moisture. The combined PDO algorithm estimates seasonal subsidence, ALT, and the vertical soil moisture profile. This study is the first in a two‐part series which describes the PDO algorithm. In this study, we present several calibration methods which were developed for the L‐band InSAR data used in the PDO algorithm. Part II describes in detail the PDO algorithm and associated data processing techniques (Chen et al., 2021, submitted to this special issue).

## 2 Background: InSAR Technique

The measured interferometric phase *δϕ* is commonly expressed (Zebker & Villasenor, 1992) as:

(1) $$\delta \phi =\frac{4\pi }{\lambda }\delta r_{los}+\delta \phi_{dem}+\delta \phi_{prop}+\delta \phi_{decor}+\delta \phi_{unw}+\delta \phi_{spat}$$

where *λ* is the radar wavelength, *δr*_*los*_, the signal of interest, is the change in range along the radar line‐of‐sight (LOS), and the subsequent terms are phase errors due to: errors in the digital elevation model (*δϕ*_*dem*_), propagational phase errors (*δϕ*_*prop*_, is itself comprised of errors due to variable tropospheric delay, platform positioning errors, and uncorrected platform motion errors), phase decorrelation (*ϕ*_*decor*_), phase unwrapping errors (*ϕ*_*unw*_), and spatial heterogeneity of surface scattering properties within the multilook window (*δϕ*_*spat*_)—due to variable soil moisture or vegetation cover, for example. Due to the very small spatial baseline (*B* <= 5 m) associated with UAVSAR interferometric products, we assume that the digital elevation model errors are small compared to other noise terms. Propagational phase errors, decorrelation phase errors, phase unwrapping errors, and errors due to the spatial heterogeneity of surface scattering properties are all present in UAVSAR interferograms and can significantly bias retrievals if not properly considered.

Typical phase errors due to variable tropospheric path delay are on the order of 1–3 cm for individual L‐band interferograms (Chen et al., 2020; Zebker et al., 1997). Tropospheric phase delays are commonly separated into stratified (systematic) delays correlated with topography and turbulent (stochastic) delays due to the time‐variant turbulence of tropospheric water content (Doin et al., 2009; Fattahi & Amelung, 2015; Jolivet et al., 2011). Correction of tropospheric noise on an individual interferogram basis is possible through the use of forward models of tropospheric water content from reanalysis datasets of sufficient quality (Jolivet et al., 2014). Averaging multiple interferograms that share a signal of interest (Chen et al., 2020; Sandwell & Price, 1998) and low‐pass filtering (Murray & Lohman, 2018) can be used to mitigate the turbulent component of tropospheric noise. In time series analyses, the stratified component of tropospheric delay can be empirically estimated through regression with a digital elevation model (Dini et al., 2019; Zebker, 2021). However, with only two sets of L‐band flights for each study site in the 2017 ABoVE airborne campaign, only a single interferogram could be formed per study site, precluding multiple‐interferogram approaches.

Decorrelation phase noise and phase unwrapping errors are both more severe where interferometric coherence is low (Agram & Simons, 2015; Zebker & Villasenor, 1992). UAVSAR interferometric products tend to exhibit interferometric coherence much higher than analogous spaceborne InSAR products; further, masking out of low‐coherence pixels is a straightforward method of culling interferometric data that are corrupted by significant decorrelation noise. Individual interferograms that contain discontinuous regions of coherent pixels separated by decorrelated pixels can often exhibit phase unwrapping ambiguities and local phase unwrapping errors, which can be mitigated by empirical unwrapping correction algorithms and masking out of statistical outliers, respectively (Benoit et al., 2019; López‐Quiroz et al., 2009; Yunjun et al., 2019).

Spatial averaging (“multilooking”) is usually performed during interferogram formation under the assumption of signal ergodicity within the multilook window. The scattering elements which give rise to radar backscatter within a multilooking window are assumed to produce an ergodic signal; that is, each pixel corresponds to a realization from the same statistical distribution. Complicated surfaces, such as playas, vegetated terrains, and tundras are characterized by a high degree of spatial variability and can exhibit signs of significant departure from ergodicity within multilook windows (Michaelides, 2020; Zwieback & Meyer, 2020). While taking additional looks is commonly employed to reduce decorrelation phase errors, such an approach has limited utility in mitigating errors due to nonergodic spatial variability of surface scatterers.

In this study, we describe several calibration techniques that we developed to remove atmospheric noise, correct phase unwrapping errors, improve absolute phase referencing methods, and quantify uncertainty due to the nonergodicity of multilooked phase. To better understand the differences between the retrieval results using airborne InSAR (PDO) and spaceborne InSAR (ReSALT), we conducted several model inversions using simulated airborne datasets to illustrate the sensitivity of retrieval results, to inherent properties of the data collection scheme used in the ABoVE airborne campaign. To better quantify sources of uncertainty in retrieving ALT from airborne InSAR measurements, we investigated the influence of the native spatial resolution of the L‐band interferometric radar on the dynamic range of inferred deformation values, as well as the limitation to a single interferogram per thaw season from which estimates of seasonal subsidence are inferred. We also develop a novel method of estimating uncertainty in both interferometric phase and measured seasonal subsidence due to nonergodic spatial variability by modeling interferometric phase as a random variable drawn from a Gaussian mixture distribution (GMD). Finally, we discuss the implications of these methods for retrieving active layer physical properties from InSAR measurements.

## 3 Study Sites

**Figure 1:**
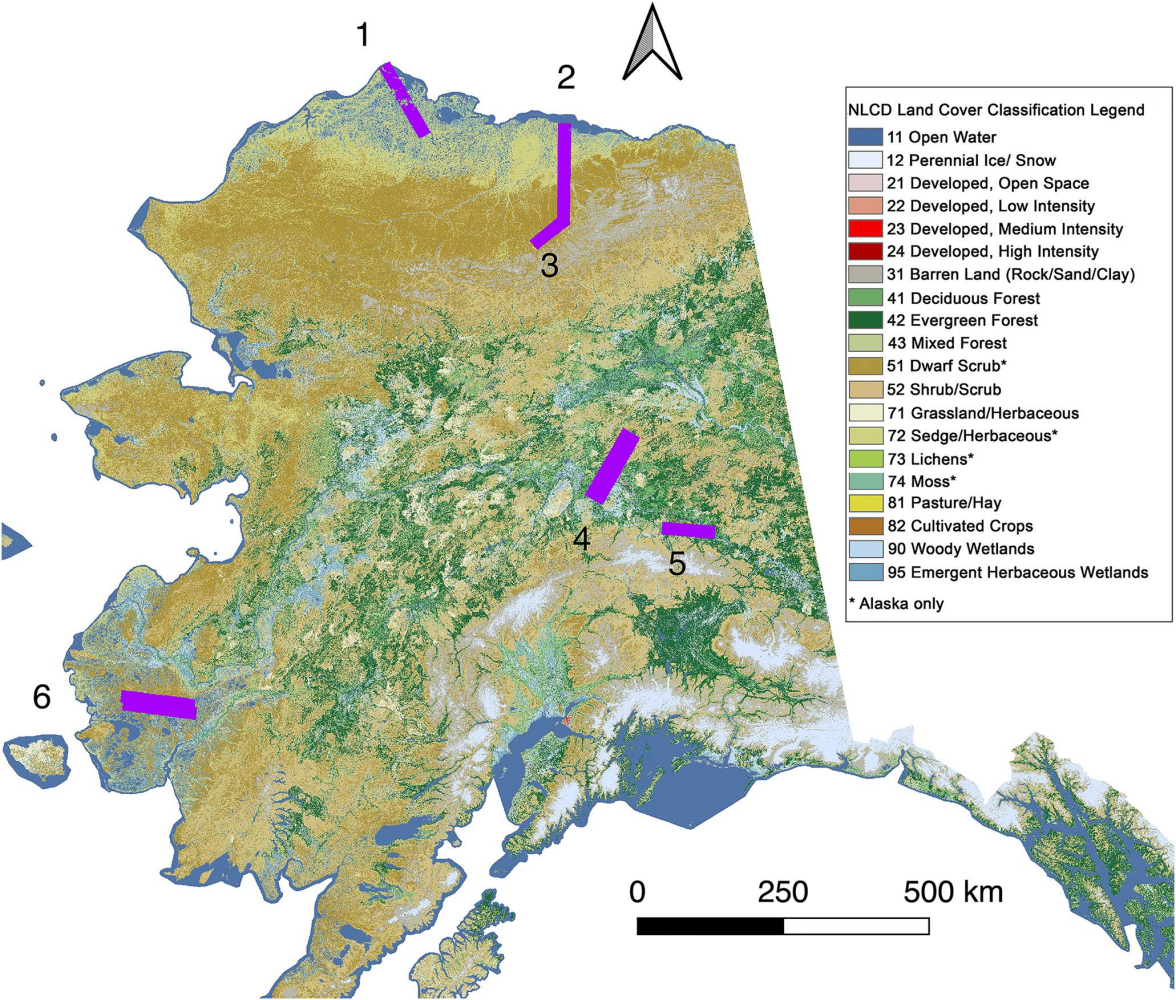
2016 National Land Cover Database map of Continental Alaska, with radar swaths corresponding to the six study sites outlined in magenta. 1: Barrow, 2: Deadhorse, 3: Toolik, 4: Bonanza Creek, 5: Delta Junction, 6: Yukon‐Kuskokwim Delta. Legend adapted from (Dewitz, 2019).

To assess the UAVSAR results presented in this study, we chose to focus on six representative swaths from the 2017 Alaskan airborne campaign. These swaths were chosen for their diversity of ecoregion, land cover, expected permafrost distribution, and availability of in situ measurements of active layer thickness and soil moisture. The location of these swaths are overlain on the Alaska National Land Cover Database 2016 in Figure 1 (Dewitz, 2019).

The Barrow, Deadhorse, and Toolik swaths all lie on the Alaskan North Slope, characterized by continuous permafrost distribution (Brown et al., 2015). The Barrow swath extends from the Arctic Ocean South through the Arctic coastal plain. Land cover over the swath is characterized by numerous open water thermokarst lakes, emergent herbaceous wetlands, and sedge and herbaceous tundra. The Deadhorse swath extends from the Arctic Ocean South toward the foothills of the Brooks Range. Along this North‐South gradient, land cover type transitions from open water thermokarst lakes and sedge/herbaceous tundra in the North into dwarf shrub and scrub tundra in the South. The Toolik swath, which is entirely within the foothills of the Brooks Range is characterized by dwarf shrub and scrub tundra.

The Bonanza Creek swaths and Delta Junction swaths lie within the discontinuous permafrost zone of Interior Alaska. Characterized by warmer annual temperatures than the North Slope, this region of Interior Alaska is dominated by coniferous forest. Both swaths are prominently characterized by evergreen and deciduous forests, woody wetlands, and isolated patches of shrub and scrub. Further, the Delta Junction swath has regions of open water and cultivated crops.

Acquired over the Izaviknek Highlands in the Yukon‐Kuskokwim (YK) Delta in Southwest Alaska, the YK Delta swaths are characterized by dwarf shrub tundra, emergent herbaceous wetlands, and open water thermokarst lakes. The YK Delta is one of the warmest areas of the Arctic tundra biome; it has higher average temperatures than both Interior Alaska and the North Slope, and lies within the discontinuous permafrost zone. The Izaviknek Highlands is notable for a complex of several fire scars dating from the 1970s until present day.

Topographic characteristics of each survey site were derived from the 30 arc second SRTM30 digital elevation model, which was used for interferometric processing of the UAVSAR data (Farr et al., 2007; Jet Propulsion Laboratory, 2013). These survey sites are characterized by modest to little topographic relief, such that topographically correlated tropospheric phase signals are assumed to be small (Doin et al., 2009; Zebker, 2021). Topographic statistics for each study site are listed in Table 1, along with the approximate dates of thaw and freeze onset, as derived from gridded Daily Surface Weather and Climatological Summaries (DAYMET) temperature reanalysis data (Thornton et al., 2016). All synthetic aperture radar (SAR) scenes were acquired within this thaw time window; accordingly snow cover is expected to be non‐existent in any SAR scenes.

**Table 1:** Summary Statistics of Six Selected Study Sites From the ABoVE Dataset

Site	Standard Deviation of Elevation (m)	Mean Slope (°)	Standard Deviation of Slope (°)	Approximate Thaw Onset	Approximate Freeze Onset
Barrow	9.85	0.11	0.22	June 14, 2017	September 29, 2017
Bonanza Creek	232.04	3.97	4.14	April 22, 2017	October 16, 2017
Deadhorse	174.98	1.11	1.35	June 6, 2017	September 24, 2017
Delta Junction	87.11	2.14	3.19	April 21, 2017	October 15, 2017
Toolik	152.34	2.93	3.26	May 11, 2017	September 19, 2017
YK Delta	9.48	0.14	0.27	April 11, 2017	October 16, 2017

## 4 Methods

### 4.1 Overview of Processing Steps

The PDO joint retrieval utilizes spatially co‐registered P‐band polarimetric SAR (∼70 cm wavelength) and L‐band InSAR (∼24 cm wavelength) datasets to simultaneously estimate the vertical profile of volumetric water content within the active layer, subsidence of the seasonally thawing active layer, and the ALT during the 2017 thaw season. The PDO joint retrieval has been applied to 66 swaths collected across the ABoVE Domain during the 2017 thaw season. Estimates of ALT, seasonal subsidence, vertical profiles of volumetric water content, and the uncertainties of each estimated parameter are provided at a 30 m spatial resolution. For further details of the PDO joint retrieval algorithm, we refer readers to Chen et al. (2021, In press).

All P‐band and L‐band data analyzed in this paper and in (Chen et al., 2021) are made available by NASA/JPL UAVSAR (https://uavsar.jpl.nasa.gov/). While the L‐band UAVSAR has a native resolution of 1.67 and 0.6 m in range and azimuth respectively, InSAR pair products are provided at a 5 m by 7.2 m (range/azimuth) spatial resolution. We applied an additional multilooking (spatial averaging) operator to the wrapped interferograms, which reduced the interferometric noise at the expense of coarsening the spatial resolution to 30 m. All InSAR products were then phase unwrapped using the SNAPHU algorithm (Chen & Zebker, 2002), and a multimodal peak detection algorithm was applied to empirically correct any phase ambiguities associated with phase unwrapping errors (Section 4.2). All pixels in the unwrapped interferogram exhibiting a coherence below 0.35 were masked out (as in Jiang and Lohman, 2021, and comparable to the choice of 0.3 in Bernhard et al., 2020), as were any remaining pixels with an unwrapped phase more than four standard deviations (SDs) from the swath‐wide mean. This masking removed decorrelated pixels associated with standing water, and residual phase unwrapping errors.

A common method of resolving three‐dimensional deformation vectors from spaceborne InSAR platforms relies on the diversity of viewing angles afforded by ascending and descending orbits (Fialko et al., 2001; Hanssen, 2001; Wright et al., 2004). A single viewing geometry in the ABoVE dataset does not allow for an unambigiuous three‐dimensional decomposition of the LOS InSAR‐derived deformation. Instead, we projected the unwrapped phase into the vertical direction using the unit vector of the radar (Chen et al., 2014) under the assumption that ground surface subsidence is predominantly in the vertical direction (Liu et al., 2010; Liu et al., 2012). Such an approximation is valid in flat areas devoid of significant topographic relief and gradients (see Section 3), where seasonal subsidence of the thawing active layer is predominantly vertical.

Propagational phase errors due to variable tropospheric phase delay, aircraft positioning errors, and residual aircraft motion were then empirically removed through the use of a high‐pass filter (Section 4.3). Interferometric measurements are fundamentally relative and must be converted into an absolute reference frame to infer physically meaningful deformation values. We applied a multi‐point calibration technique to simultaneously convert interferometric phase into absolute deformation measurements and mitigate the larger measurement uncertainties inherent to a single‐interferogram inversion (Section 4.5). Finally, we quantified the component of interferometric measurement uncertainty due to the fine scale (<30 m) spatial variability exhibited by tundra and periglacial surfaces (Section 4.6).

### 4.2 Multimodal Phase Unwrapping Ambiguity Correction

When lakes, rivers, or other features which do not maintain interferometric coherence divide the SAR image, phase unwrapping can produce spatial discontinuities in unwrapped phase, and therefore relative deformation. The fundamental measurement of SAR interferometry—a change in phase between two image acquisitions—is directly proportional to the change in range from the radar instrument to the ground resolution element. However, because interferometric phases are phasor terms, they are cyclically 2*π* ambiguous, or “wrapped” (Goldstein et al., 1988). In order to convert an interferogram into an estimate of a true deformation field, the wrapped phase values must be converted into total changes in range, that is, “unwrapped”. Phase unwrapping can often result in phase ambiguities, where portions of the unwrapped image are discontinuous from the rest of the image by an integer number of 2*π* phase signals (Yunjun et al., 2019). If not corrected, phase unwrapping errors can yield physically unrealistic, spatially isolated estimates of deformation. Where three or more co‐registered interferograms are available, phase closure constraints can be used to correct for unwrapping errors (as in Benoit et al., 2019; Yunjun et al., 2019). With only a single interferogram per study site, such an analysis is not possible in this study. After phase unwrapping, we performed a multimodal peak detection algorithm on the histogram of unwrapped phase values. Any secondary peak modes in the histogram were re‐referenced to the primary histogram peak by the best‐fitting integer number of 2*π* phase cycles, yielding a unimodal histogram of unambiguous, unwrapped phase.

### 4.3 Removal of Propagational Phase Errors

When significant spatial or temporal variations in tropospheric water vapor content, temperature, and pressure are present in individual SAR images, phase screens can manifest in interferometric products, which can considerably bias estimates of ground deformation (Zebker et al., 1997). Similarly, imprecise platform position information and unmitigated platform motion can both introduce propagational phase screens that can complicate the retrieval of ground surface deformation (Fattahi & Amelung, 2014; Stevens et al., 1995; Zebker et al., 2010). Some past studies of permafrost dynamics with multiple interferograms simply discarded those contaminated with severe propagational phase noise (Jingyi et al., 2017; Liu et al., 2010). Where sufficient weather reanalysis or global positioning system soundings are available, turbulent atmospheric noise can be removed from individual interferograms (Dini et al., 2019; Jolivet et al., 2014; Jolivet et al., 2011), and characterized through spectral filtering and geostatistical methods (Murray et al., 2019). Digital elevation models can be employed to empirically remove the stratified component of tropospheric noise during time series analysis of interferogram networks (Dini et al., 2019; Zebker, 2021), and data redundancy during time series analysis can further introduce temporal smoothing of tropospheric phase errors. The single‐interferogram collection strategy of the 2017 ABoVE airborne campaign prevented these previous techniques of tropospheric error mitigation, necessitating a method of mitigating any propagational phase errors directly from the interferogram.

Initial noise removal attempts using atmospheric reanalysis data to estimate tropospheric phase delays (Jolivet et al., 2014, 2011) were unsuccessful due to the low density of weather stations over the ABoVE domain, which prevented accurate estimation of tropospheric water content at the spatial scale of collected interferograms. Instead, we applied a high‐pass filter to remove phase signals at length scales on the order of the correlation length of atmospheric humidity. This high‐pass filter exploits the large difference in correlation lengths between atmospheric phase screens (∼km, Lohman & Simons, 2005) and surface features associated with permafrost thaw (∼m, Schaefer et al., 2015). Comparable to previous studies, we chose a 15‐km threshold (Emardson et al., 2003; Foster et al., 2006; Murray et al., 2019), whereby the low‐pass phase screen was taken to be the atmospheric phase screen and was subtracted from the original unwrapped phase. We then applied a low‐pass moving filter with a 15 × 15 km Gaussian kernel to estimate the atmospheric phase screen, and the atmospheric phase screen was then subtracted from the original unwrapped interferogram, leaving behind the high‐pass signal associated with surface deformation. Due to the low topographic relief over the sites described in this study (see Section 3), we assumed that the stratified component of tropospheric noise is minimal, and that the turbulent component dominates.

### 4.4 Degree Day Correction

The ideal interferogram with which to estimate ALT would be derived from SAR scenes spanning the entire thaw season, with the first scene acquired when the active layer starts to thaw, and the second scene acquired when the active layer begins to refreeze. As the UAVSAR scene pairs do not span the entire thaw season, they do not measure the total seasonal subsidence. To estimate total seasonal subsidence, we applied a modified form of the deformation scaling method based on Stefan's law and first introduced in Liu et al. (2010):

(2) $$S=\frac{\sqrt{ADDT_{max}}}{\sqrt{ADDT_{2}-ADDT_{1}}}\Delta \delta$$

where *S* is the total seasonal subsidence, *ADDT*_*max*_ is the maximum accumulated degree days of thaw (ADDT) for a given thaw season, *ADDT*_1_ and *ADDT*_2_ are the ADDT values for the first and second SAR acquisition times, respectively, and Δ*δ* is the measured deformation between the two SAR acquisitions (see Appendix A for a complete derivation of Equation 2).

Equation 2 can be simplified to the form:

(3) S=αΔδ

where the dependence on ADDT is encapsulated in the ADDT factor *α*. The ADDT factor can then be calculated at each study site from gridded DAYMET temperature reanalysis data (Thornton et al., 2016) and used to estimate the total seasonal subsidence from the measured interferometric deformation.

### 4.5 Multi‐Point Phase Referencing

After phase unwrapping and atmospheric noise removal, relative unwrapped deformation values must be converted into absolute deformation values. This is commonly done by identifying pixels where independent estimates of surface deformation are available, such as through global positioning system stations (Chen et al., 2014). When independent data is not readily available, phase referencing is commonly done by selecting a pixel where surface deformation is expected to be near‐zero, such as exposed bedrock or gravel floodplains on the Alaskan North Slope (Liu et al., 2010; Liu et al., 2012). Where in situ measurements of thaw depth and active layer thickness are available, they have been used to forward model an associated surface subsidence, which can then be used as a calibration point to convert relative deformation to absolute deformation values (Michaelides, Schaefer, et al., 2019; Schaefer et al., 2015). All of these calibration techniques are ‘single‐point' calibration methods, meaning they shift the scene‐wide mean phase value for each interferogram by adding the same phase value to every pixel in an interferogram.

The lack of reliable in situ observations with which to calibrate interferometric phase over most of the ABoVE domain necessitated a data‐driven approach to phase referencing. We adapted the technique originally used in Liu et al. (2010), which referenced InSAR data over the Alaskan North Slope with respect to the largest observed phase within the interferogram above a certain coherence threshold. We employ a more conservative approach, referencing each interferogram to the 95th percentile of scenewide unwrapped phase. This value was chosen due to the observation that where in situ observations were available for phase referencing, 95% of the interferogram exhibited absolute phase values greater than zero.

The UAVSAR unwrapped interferograms are characterized by a larger dynamic range of observed deformation values than spaceborne InSAR studies of permafrost subsidence. This can be partially attributable to residual propagational phase noise (such as nonlinear tropospheric noise), the difference in native spatial resolution between airborne and spaceborne SAR platforms, and the lack of multiple interferograms with which to constrain the estimated seasonal subsidence of the active layer. These latter phenomena are discussed in greater detail in Sections 6.2.1, 6.2.2.

**Figure 2:**
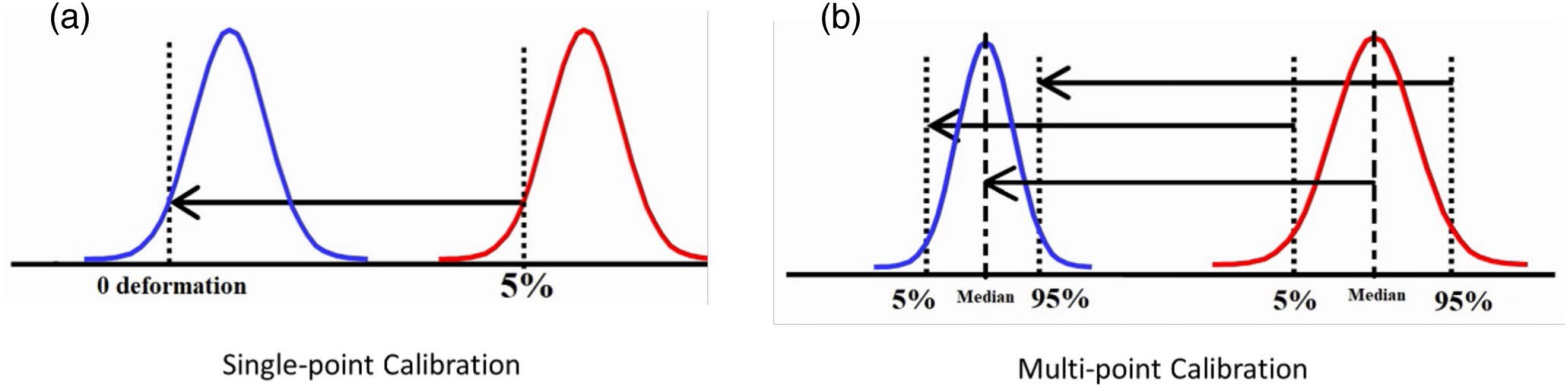
(a) The single‐point calibration technique. (b) The multi‐point calibration technique. Red indicates the uncalibrated, relative deformations, blue indicates calibrated, absolute deformations, and vertical black dotted lines indicate known values chosen to calibrate the distribution of phase values.

To mitigate the impact of this larger dynamic range of observed deformation values on the joint retrieval of ALT and soil moisture, we implemented a “multi‐point calibration” model that simultaneously converted relative InSAR measurements to absolute measurements and rescaled the distribution of deformation measurements, as conceptually shown in Figure 2. This multi‐point calibration method yielded a dynamic range of inferred deformation and ALT that is consistent with independent in situ measurements of soil thaw depth, ALT, and moisture measurements collected at regions across the ABoVE domain. Relative interferometric measurements were calibrated with in situ data:

(4) $$\frac{\delta_{sar}-\delta_{sar,median}}{\delta_{sar,95\%}-\delta_{sar,5\%}}=\frac{\alpha \delta_{sar,cal}-\delta_{insitu,median}}{\delta_{insitu,95\%}-\delta_{insitu,5\%}}$$

where *δ*_*sar*_ is UAVSAR deformation measurements, *δ*_*insitu*_ is estimated in situ deformation values, and $\delta_{sar_{c}al}$ is the UAVSAR deformation measurements that have been subjected to the multi‐point calibration. The subscripts “median”, “95*%,*” and “5*%*” correspond to the corresponding percentiles of distribution‐wide deformation values, and *α* is the ADDT factor, an empirically determined coefficient used to scale measured subsidence into an estimate of total seasonal subsidence, as in Section 4.4. Rearranging Equation 4 and expressing it in terms of the calibrated UAVSAR deformation yields:

(5) $$\delta_{sar_{c}al}=\frac{\delta_{insitu,95\%}-\delta_{insitu,5\%}}{\delta_{sar,95\%}-\delta_{sar,5\%}}\cdot \frac{\delta_{sar}}{\alpha }+\frac{1}{\alpha }\cdot \left(\delta_{insitu,median}-\left(\frac{\delta_{insitu,95\%}-\delta_{insitu,5\%}}{\delta_{sar,95\%}-\delta_{sar,5\%}}\right)\cdot \delta_{sar,median}\right)$$

which can be simplified to:

(6) $$\delta_{sub}=\alpha \delta_{sar_{c}al}=\alpha \left(a\delta_{sar}+b\right)$$

where *δ*_*sub*_ is the estimated seasonal subsidence and

(7) $$a=\frac{\delta_{insitu,95\%}-\delta_{insitu,5\%}}{\delta_{sar,95\%}-\delta_{sar,5\%}}$$

(8) $$b=\delta_{insitu,median}-\left(\frac{\delta_{insitu,95\%}-\delta_{insitu,5\%}}{\delta_{sar,95\%}-\delta_{sar,5\%}}\right)\cdot \delta_{sar,median}$$

Inspection of Equation 6 illustrates that this multipoint calibration technique contains both a scene‐wide shift (*b* Equation 8 term; analogs to single‐point absolute phase referencing techniques), and a scaling of the scene‐wide distribution (*a* term Equation 7). This multi‐point calibration technique is a linear operator on the scene‐wide deformation estimate, whereas single‐point calibration techniques apply a constant shift to the scene.

We assessed the robustness of the calibration method via a bootstrapping strategy, whereby 10% of all in situ reference points were randomly selected and withheld from the calibration and used for validation; this process was then repeated 10,000 times. We define the calibration error as:

(9) $$error=\frac{1}{N}\sum_{n=1}^{N}|\delta_{sar_{c}al}-\delta_{insitu}|$$

In situ reference data consisted of colocated mechanical probing and ground penetrating radar measurements of thaw depth, which were converted into corresponding estimates of surface deformation using the ReSALT model (Liu et al., 2012; Schaefer et al., 2015). The sampling strategy and locations of collected reference data are described in greater detail by Clayton et al. (2021).

### 4.6 Interferometric Uncertainty Calculation

Previous InSAR‐based studies of permafrost dynamics have empirically calculated deformation uncertainty from the root mean square error between the measured deformation time series and the ADDT model‐based deformation time series (Schaefer et al., 2015). This “data‐model residuals” approach requires an over‐determined system of equations, that is, several interferograms. As previous studies have relied on spaceborne InSAR platforms, repeat observations are readily available; the “single interferogram per site” collection strategy of the ABoVE UAVSAR campaign, however, precluded such an analysis. Instead, we estimated uncertainty in deformation estimates directly from the statistics of the interferometric signal. The Cramer‐Rao bound for phase variance is a commonly reported metric in the InSAR community (Guarnieri & Tebaldini, 2007; Tough et al., 1995). However, in SAR images that have been heavily multilooked (as in UAVSAR images), the Cramer‐Rao bound yields an estimate of phase variance that is much smaller than the 1–3 cm errors commonly encountered in individual interferograms due to atmospheric noise (Zebker et al., 1997). Further, nonergodicity of the interferometric phase within a multilook window can result in actual uncertainties much larger than the Cramer‐Rao lower bound. Additionally, even if propagational phase errors are properly mitigated (as we implicitly assumed in this analysis), permafrost regions exhibit a high degree of spatial variability at scales finer than the SAR resolution due to the factors that control ALT (e.g., heterogeneous vegetation cover, surface hydrology, ground thermal regime, subsidence of the ground surface), such that root mean square errors of at least 0.5–2 cm are commonly encountered in time series analysis in permafrost regions (Michaelides, Schaefer, et al., 2019; Schaefer et al., 2015; Zwieback & Meyer, 2020).

**Figure 3:**
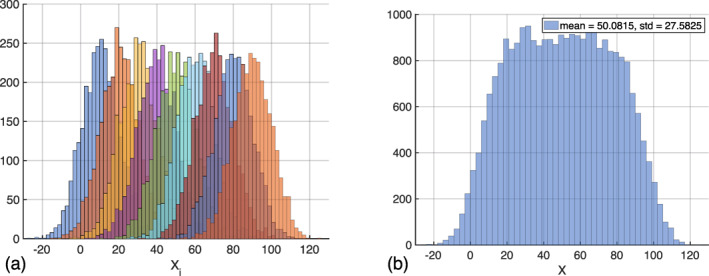
Representative histogram for a random variable drawn from a GMD with nine subpopulations with different mean values. While each subpopulation has similar variances, the variability in the mean of each subpopulation (a) results in a histogram (b) that diverges from Gaussianity and exhibits a larger standard deviation and mean absolute deviation than any of its subpopulations.

Under the assumption that propagational phase errors have been removed from interferograms as in Section 4.3, we wished to quantify the interferometric error associated with nonergodicity within the multilook window, which is due to fine‐scale spatial variability of the tundra surface. To account for the high degree of spatial variability across the Arctic tundra, we modeled the interferometric phase measured in a single UAVSAR interferogram as a random variable drawn from a GMD (see Figure 3). Gaussian mixture modeling is a probabilistic method of expressing subpopulation heterogeneity within a sampled distribution without explicitly defining the statistics of subpopulations (Moraru et al., 2019). It has been used in image classification, segmentation, and discrimination (Paalanen et al., 2006; Xia et al., 2016), MRI imaging texture classification (Moraru et al., 2019), and target tracking (Trailovic & Pao, 2002). The variance of a GMD random variable is a linear combination of the individual variances of the Gaussian subpopulations averaged into the GMD, as well as the variance of the individual means of the same Gaussian subpopulations (Hasselblad, 1966). Within the framework of interferometric images, the phase variance of a multilooked pixel is the quadratic sum of the Cramer‐Rao phase variance of each pixel that is averaged into the final multilooked pixel and the variance of the mean phase values of all pixels that are averaged together within a multilook window:

(10) $$\sigma_{ml}^{2}=\frac{1}{N}\sum_{n=1}^{N}\sigma {cr,i}^{2}+E\left[\mu_{i}^{2}-\mu_{ml}^{2}\right]$$

where *σ*_*m*_ *l* is the SD of the interferometric phase of the multilooked pixel, *N* is the number of pixels averaged together during interferogram formation, *E*[*x*] is the expectation of *x*, *σ*_*cr*,*i*_ and *μ*_*i*_ are the *i*th Cramer‐Rao phase SD and the mean interferometric phase for the *i*th pixel in the multilooking operation, respectively, and *μ*_*ml*_ is the mean interferometric phase of the multilooked pixel.

## 5 Results

### 5.1 Multimodal Phase Unwrapping Ambiguity Correction

**Figure 4:**
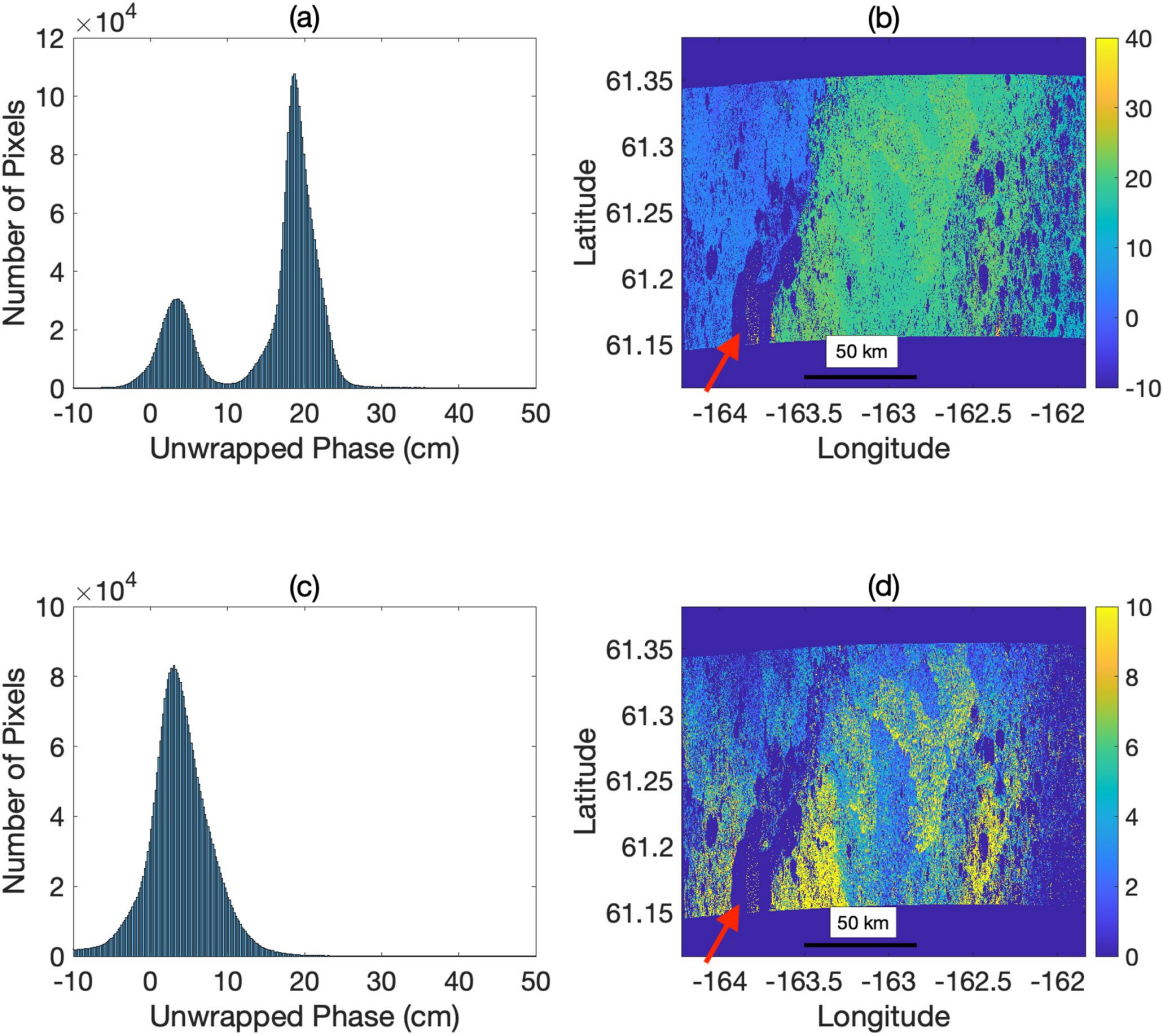
The multimodal correction removes phase ambiguities that arise during phase unwrapping. The phase ambiguity is clearly evident in the histogram of values (a) as well as the image (b); the left half of the image is offset from the rest of the image by an integer number of 2*π* phase cycles, resulting in a multimodal histogram of phase values. After applying the multimodal correction, The entire image is calibrated to the same 2*π* phase cycle, resulting in a unimodal histogram of phase values (c), and a physically realistic deformation signature (d).

Applying the multimodal correction, described in Section 4.2, successfully corrects any major areas of 2*π*‐ambiguous unwrapped phase. This is illustrated in Figure 4 for a swath of the YK Delta. The original unwrapped phase exhibited a clear discontinuity between the western and eastern halves of the swath due to Aropuk Lake (indicated by a red arrow). The multimodal correction successfully classifies pixels associated with the two discontinuous regions and applies the necessary 2*π* integer‐multiple to the western half of the scene so that the entire scene is 2*π* contiguous. The phase unwrapping ambiguity correction presented here shares similarities with phase closure‐based methods, although our proposed method is considerably simpler and less sophisticated due to the lack of a traditional network of repeat pass interferograms, precluding the option of imposing phase closure constraints.

### 5.2 Removal of Propagational Phase Errors

**Figure 5:**
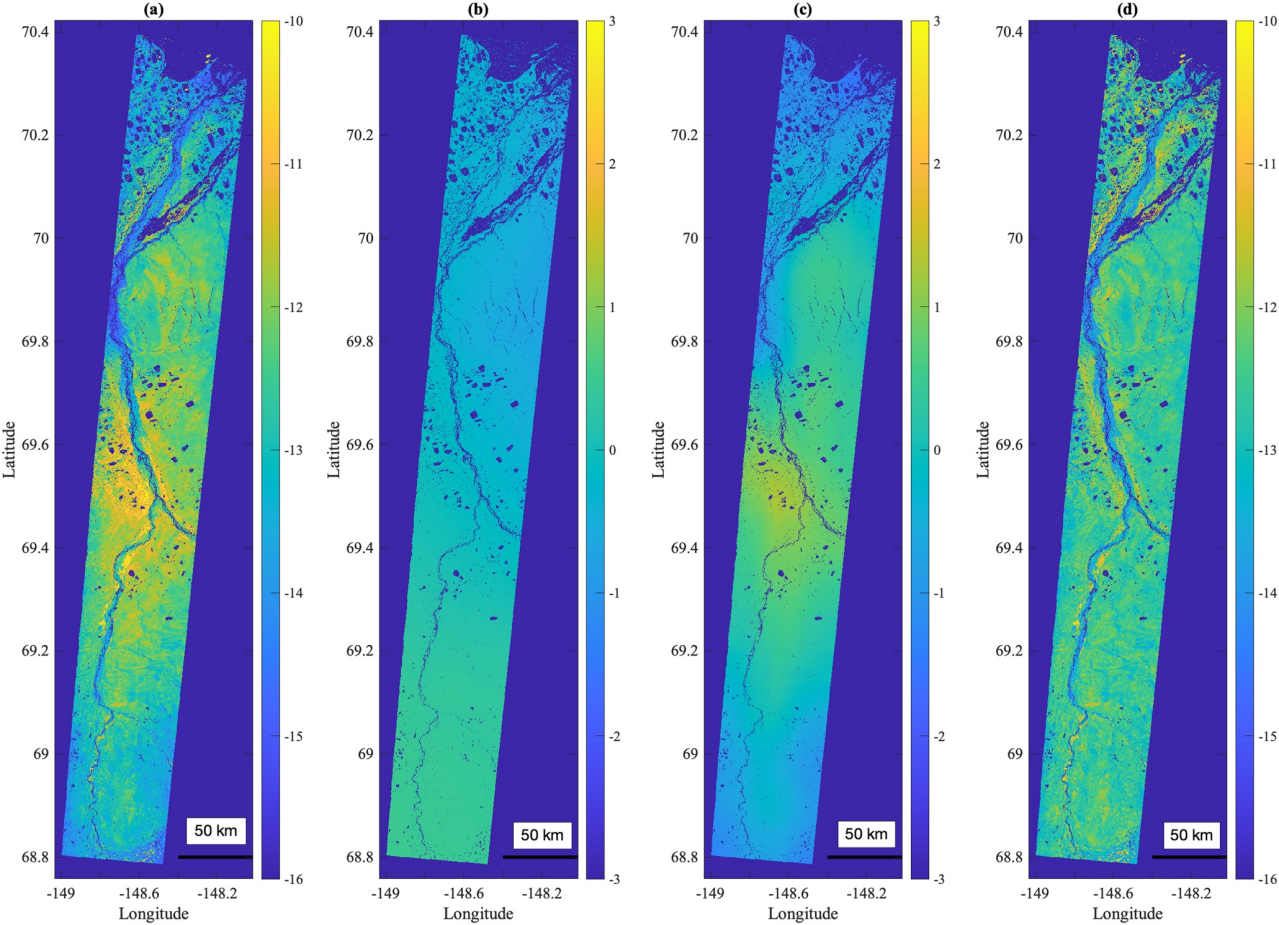
(a) Original unwrapped phase for Deadhorse swath. (b) Tropospheric phase delay inferred from ECMWF. (c) Inferred propagational phase from low‐pass filter. (d): Inferred surface deformation due to active layer thaw (high‐pass spatial component).

Figure 5 illustrates the result of estimating propagational phase errors via a low pass filter. The original unwrapped phase (left panel) exhibits a phase signal that contains both low frequency and high frequency components. Applying a Gaussian filter to the original data results in a low pass estimate of the low frequency component, which is assumed to represent propagational errors associated with tropospheric path delay, uncompensated aircraft motion, and any aircraft positioning errors (middle panel). This low frequency component exhibits an approximately quadratic behavior as a function of latitude and is unlikely to be associated with deformation of the thawing active layer. After removal of the low pass phase signal, the high pass corrected exhibits a phase signal more consistent with local topography and surface hydrology (right panel).

This method relies on the assumption that propagational phase errors and surface subsidence terms have frequency components that are sufficiently separable by a simple frequency pass band approach. Care must be taken when designing such a filtering approach such that the deformation signal of interest is not partially removed (which can occur if the deformation signal has a characteristic length scale greater than the 15 km threshold chosen in this study), and the presence of residual propagational phase errors is minimized.

### 5.3 Multi‐Point Phase Referencing Results

**Figure 6:**
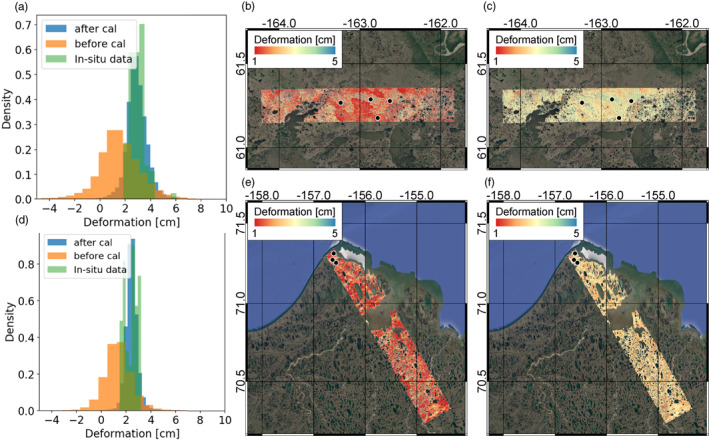
Comparison between relative and calibrated deformation distribution over the Yukon‐Kuskokwim (YK) Delta and Barrow study sites. Scenewide histogram of deformation values for YK Delta (a), and Barrow (d); InSAR‐derived surface deformation before calibration for YK Delta (b), and Barrow (e); and InSAR‐derived surface deformation after calibration for YK Delta (c) and Barrow (f). Locations of all in situ measurements are denoted by black dots.

**Figure 7:**
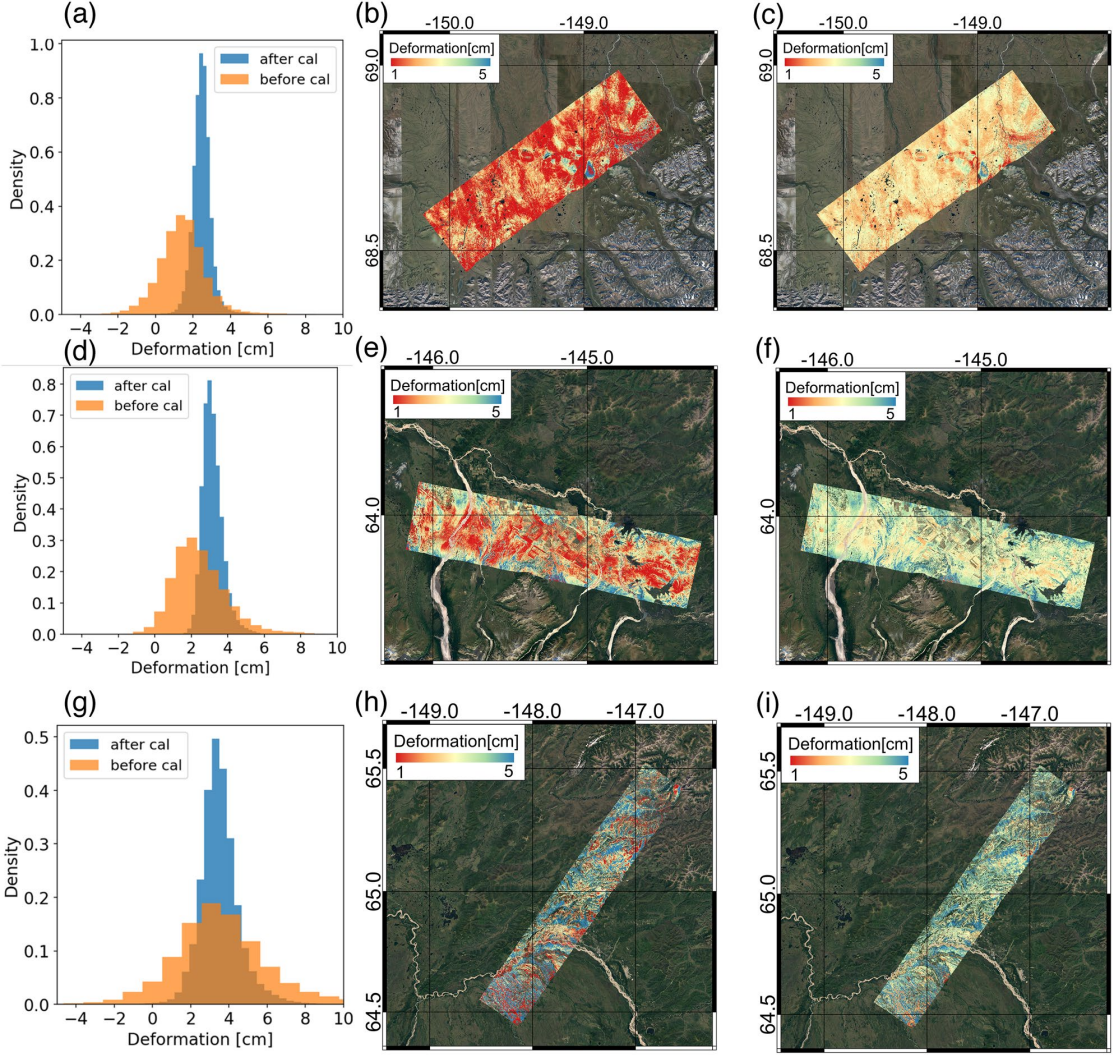
Comparison between relative and calibrated deformation distribution over Toolik Lake, Delta Junction, and Bonanza Creek study sites. scenewide histogram of deformation values at Toolik (a), Delta Junction (d), and Bonanza Creek (g); InSAR‐derived surface deformation before calibration at Toolik (b), Delta Junction (e), and Bonanza Creek (h); and InSAR‐derived surface deformation after calibration at Toolik (c), Delta Junction (f), and Bonanza Creek (i).

The results of applying the multi‐point phase calibration method to the study swaths from the UAVSAR dataset are shown in Figures 6 and 7. A narrowing of the original histogram of deformation values for the Barrow and YK Deltas lines can be clearly seen; the distribution of the in situ data estimates of subsidence are in much closer agreement with the distribution of the calibrated deformation rather than the uncalibrated deformation. The multi‐point calibration method reduces the root mean square error of retrieved ALT and soil moisture for Barrow by 40% and 20%, respectively. In the YK Delta, increased subsidence is observed over the 2015 fire scars compared to their unburned surroundings, as with older fires in the region observed by Michaelides, Schaefer, et al. (2019).

The similarity in calibration factors (Table 2) for the Barrow and YK Delta swaths—located at disparate parts of the ABoVE Domain—suggests that a multi‐point calibration based upon the ABoVE soil thaw depth, ALT, and moisture dataset is applicable across much of the ABoVE Domain. To that end, we generalized the multi‐point calibration method by applying the averaged calibration factors of Barrow and YK Delta to the remaining study swaths across the ABoVE Domain. The calibrated deformation distributions for the Toolik, Delta Junction, and Bonanza Creek swaths are displayed in Figure 7, and their corresponding statistics are summarized in Table 3.

**Table 2:** Calibration Results for Barrow and YK Delta Swaths

With Bootstrapping	*a*	*b*	*α*, ADDT Factor	Regression Function *y* = *α*(*ax* + *b*)	Mean After Calibration (cm)	Overall Error (cm)
Barrow	0.386	1.85	1.0355	*y* = 0.40 + 1.92	2.46	0.65
YK Delta	0.375	1.68	1.2914	*y* = 0.48 + 2.17	2.92	0.67

**Table 3:** Calibration Results for Toolik, Delta Junction, and Bonanza Creek

With Bootstrapping	*a*	*b*	*α*, ADDT Factor	Regression Function *y* = *α*(*ax* + *b*)	Mean After Calibration (cm)	Overall Error (cm)
Toolik	0.38	1.76	1.094	*y* = 0.42 + 1.95	2.52	0.99
Delta Junction	0.38	1.76	1.27	*y* = 0.48 + 2.26	3.43	1.66
Bonanza Creek	0.38	1.76	1.21	*y* = 0.45 + 2.15	3.81	1.47

The calibrated deformation from the Toolik swath displays a correlation between ALT and topography, with well‐drained ridgetops exhibiting shallower ALT than poorly drained low lands (Chen et al., 2020). A similar relationship is observed on the Eastern end of the Delta Junction swath, as is a prominent phase signal associated with agricultural fields and likely due to a change in soil moisture or the bound moisture content of agricultural crops (Zwieback et al., 2015). The Bonanza Creek swath is characterized by more significant interferometric decorrelation, and the prominent forest cover complicates the interpretation of any phase signals observed over the extensive forest cover in the region. A more rigorous statistical analysis of these multi‐point phase calibration results is the subject of a forthcoming paper.

### 5.4 Uncertainty Results

**Figure 8:**
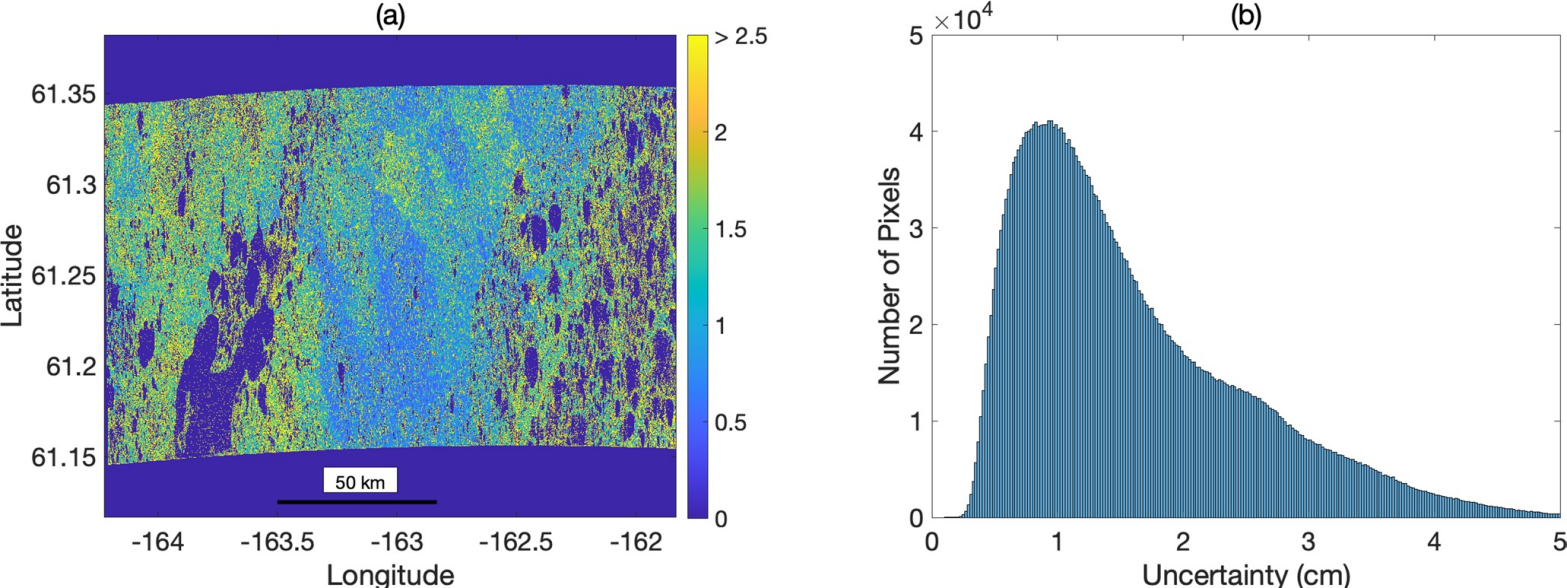
(a) Deformation uncertainty calculated assuming interferometric phase exhibits a Gaussian Mixture Distribution. Magnitude of uncertainty depends upon both the interferometric coherence as well as the local variance of interferometric phase within the 30 m multilook averaging window. (b) Scene‐wide histogram of deformation uncertainty.

We display the calculated interferometric deformation uncertainty associated with signal nonergodicity for the YK Delta swath using Equation 10 in Figure 8. Use of the GMD model for estimating deformation uncertainty results in a realistic range of uncertainty values, with areas of notable signal decorrelation exhibiting larger deformation SDs, and coherent regions exhibiting low uncertainties. The spatial patterns of estimated deformation uncertainty are consistent with independent estimates empirically derived from a data‐model residuals approach (Michaelides, Schaefer, et al., 2019). In the limit of perfect signal ergodicity during pixel multilooking (which is an implicit simplifying assumption made during multilooking) the phase variance is mathematically equivalent to the Cramer‐Rao bound. However, by accounting for nonergodicity (through the spatial variability of the mean phase in each pixel during the multilooking operation) we derive an estimate of deformation uncertainty which fully captures both decorrelation of the interferometric signal as well as the fine‐scale heterogeneity of the tundra surface. Due to the flexible implementation of this uncertainty estimation, it is fully compatible with adaptive multilooking strategies, which may further reduce phase uncertainties (Parizzi & Brcic, 2011).

## 6 Discussion

While processing techniques and calibration methods can be applied to airborne InSAR products to reduce error sources and mitigate the lack of repeat pass observational strategies, model uncertainties and residual error sources associated with the specific observational nature of airborne InSAR can introduce uncertainties during the estimation of ALT from measured deformation. Several sources of uncertainty in ALT estimation are discussed below.

### 6.1 Choice of ADDT Scaling Method

The specifics of SAR acquisition times during the thaw season determine whether the Liu et al. (2012) ADDT model or the model proposed in Section 4.4 will introduce smaller model errors while extrapolating the total seasonal subsidence from discrete deformation measurements (Xu et al., 2021, submitted to this special issue). In a situation where many redundant observations are available over a given thaw season (or several thaw seasons) and/or reliable temperature data is available, the Liu model may be more suitable. In the absence of reliable temperature data, or when few observations over a given thaw season are available, the proposed model, which is more conservative than the Liu model, can yield a more realistic estimate of surface subsidence due to permafrost thaw. This is particularly true if one observation is made during late summer, when the thaw front is near its deepest extent. Finally, neither the Liu model (Liu et al., 2012) nor the model presented here explicitly consider late autumn refreeze and uplift of the active layer, which has been observed by both Global navigation satellite system and InSAR (Chen et al., 2020; Yanagiya & Furuya, 2020; Yufeng et al., 2018). When relying on SAR scenes acquired during autumn refreeze (unlike in the 2017 ABoVE campaign), an explicit treatment of surface uplift may also be necessary.

### 6.2 Residual Contributions to ALT Uncertainty

#### 6.2.1 Effect of Spatial Resolution on Measured Phase

One of the most salient differences between the UAVSAR L‐band InSAR and comparable spaceborne InSAR instruments is the fine native spatial resolution of the UAVSAR radar. Operating at a bandwidth of 80 MHz, the UAVSAR radar has a native singlelook complex range resolution of 1.67 m and a 0.6 m azimuth resolution. The native resolutions of comparable spaceborne InSAR instruments such as Sentinel‐1A (5 m range, 20 m azimuth) and ALOS‐2 (10 m range, 5 m azimuth) can be up to an order of magnitude coarser in range and azimuth.

UAVSAR InSAR pair products are provided at a 5 m by 7.2 m (range/azimuth) spatial resolution (multilooked 3 × 12 in range/azimuth). To investigate the effect of spatial resolution, we regenerated the pair products by taking an additional 1–16 looks, yielding interferometric data at spatial resolutions of 5 m by 7.2 m to 80 m by 115 m. All interferograms were then unwrapped using the SNAPHU algorithm (Chen & Zebker, 2002) and the multimodal correction (described in Section 4.2) is applied to correct for any phase unwrapping ambiguities. We masked out decorrelated pixels (coherence less than 0.35) and referenced the data to the scene‐wide 5th percentile phase value (as in Section 4.5). SAR‐measured deformation were then converted to an estimate of ALT using the ReSALT algorithm assuming a fully saturated active layer (Liu et al., 2012).

**Figure 9:**
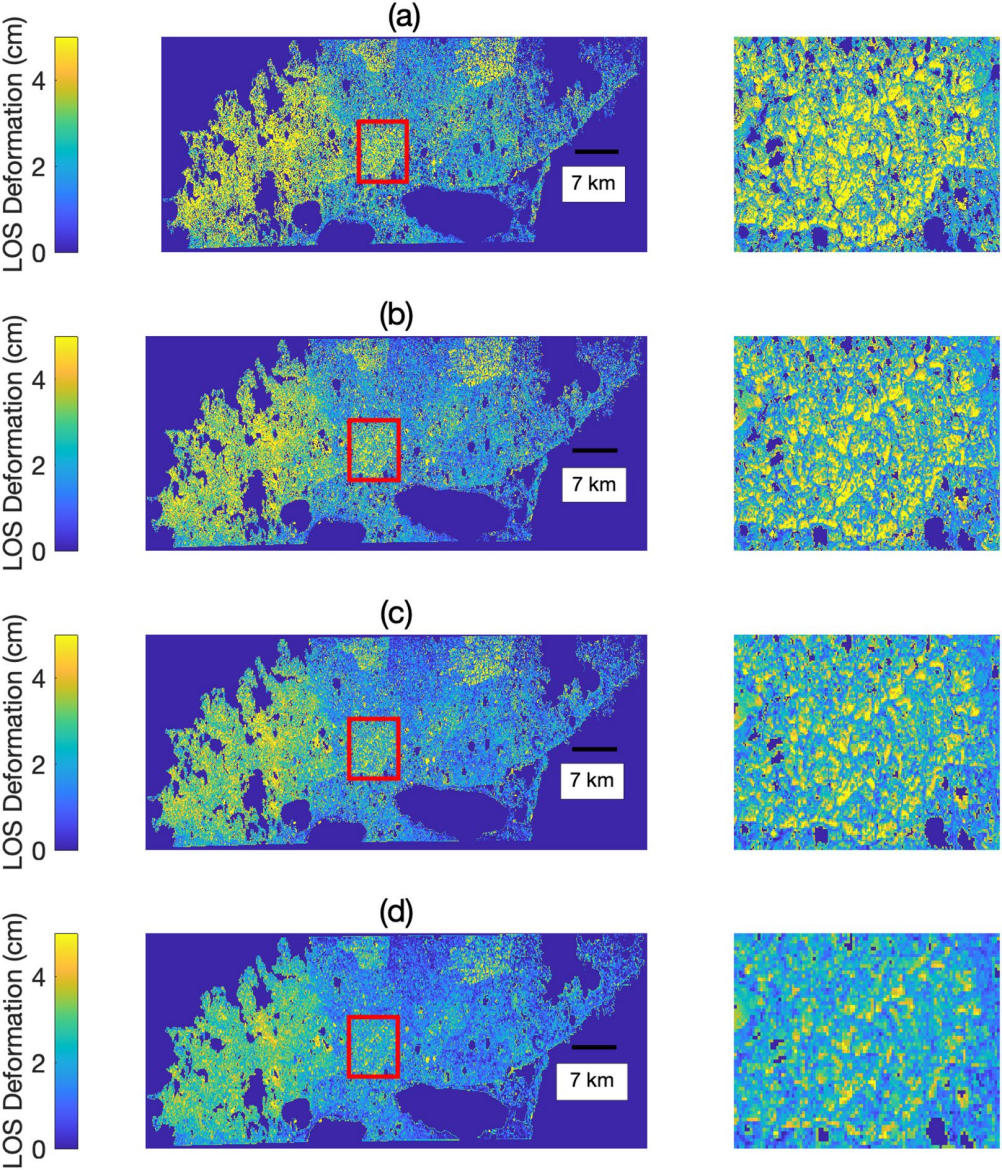
Estimated ALT in the YK Delta as a function of the spatial resolution of the interferometric data used to measure seasonal subsidence. (a) 5 m resolution; (b) 20 m resolution; (c) 40 m resolution; and (d) 80 m resolution. As spatial resolution degrades (and approaches spaceborne resolutions), the LOS deformation exhibits less fine‐scale spatial variability (see 7 × 7 km, inset at right).

**Figure 10:**
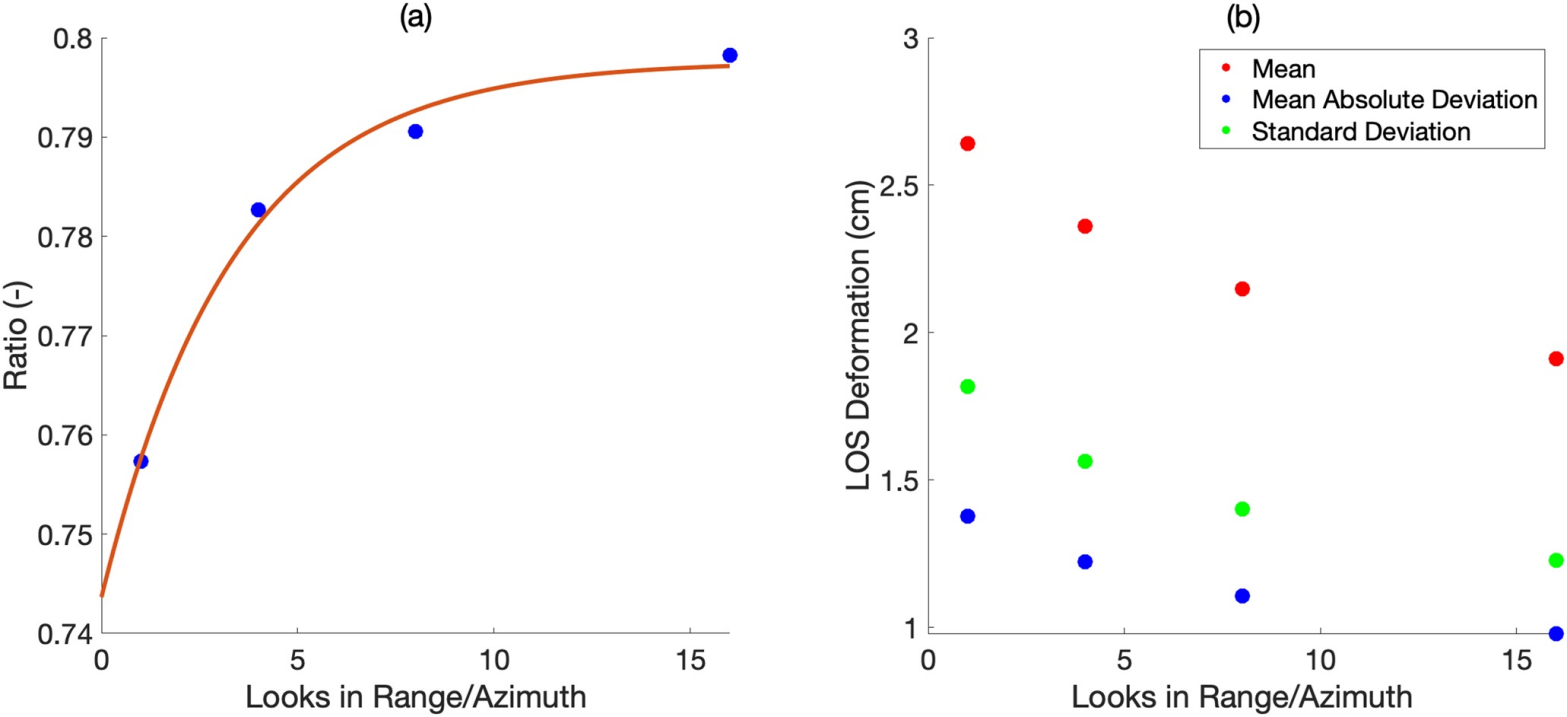
(a) Ratio of mean absolute deviation (MAD) to standard deviation (SD) of the unwrapped phase versus number of looks in range/azimuth. (b) Mean, MAD, and SD versus number of looks in range/azimuth. As the spatial resolution becomes finer, the kurtosis of the distribution increases, and the mean, MAD, SD, and MAD/SD ratio of phase all increase.

The effect of degrading the spatial resolution of interferometric data is illustrated in Figures 9 and 10. Visual analysis of the LOS deformation from the YK Delta as a function of the spatial resolution reveals that as the resolution coarsens, the ability to capture fine‐scale spatial heterogeneity worsens (see Figure 9, inset). Multilooking is commonly performed under the assumption that sufficient incoherent averaging will yield a reduction in the SD of phase values, as well as an increasingly more precise estimate of the mean phase. Interestingly, while SD and absolute deviation both decrease with increasing spatial resolution, the mean also exhibits a proportional decrease in value. A possible explanation may be that the interferometric phase is not drawn from a spatially ergodic Gaussian distribution. The departure of the phase distribution from a pure Gaussian distribution is further suggested by analyzing the ratio of mean absolute deviation (MAD) to SD of the LOS unwrapped phase (Figure 10, left panel). As *MAD*(*x*) ≤ *SD*(*x*), MAD/SD cannot exceed 1; the smaller the value of MAD/SD, the higher the kurtosis, or “tailedness” of the statistical distribution. For a Gaussian distribution, $MAD/SD=\sqrt{\frac{2}{pi}}=0.7979$. For fine native resolution, we can see that the phase is not normally distributed and exhibits a higher kurtosis than a Gaussian distribution. As the spatial resolution coarsens, MAD/SD approaches that of a Gaussian distribution.

There are several implications of these statistical observations. First, the high degree of spatial variability across permafrost regions displays a scale‐dependence which complicates direct comparison of airborne and spaceborne InSAR datasets acquired at very different native resolutions, as noted in (Xu et al., 2021, submitted to this special issue). Second, at the very fine native resolution of the UAVSAR L‐band radar, interferometric phase values do not appear to obey Gaussian statistics, implying that matching airborne resolutions to spaceborne resolutions through incoherent averaging may not sufficiently address the scale‐dependence of variability observed in Arctic regions. While the interferometric phase values approach normality as the resolution is degraded, at the 30 m spatial resolution selected for the data products generated from the PDO joint retrieval, interferometric phase still exhibits a degree of kurtosis, which can complicate ALT estimates when only a single interferogram is available. A recent study demonstrated that the choice of temporal baseline in InSAR time series analysis can similarly introduce interferometric phase biases (Ansari et al., 2020). Much like this counterintuitive result, the choice of spatial resolution may similarly introduce phase biases into both individual interferograms and time series analyses, necessitating further research into interferometric phase biases which are not directly related to geophysical deformation.

#### 6.2.2 Effect of Number of Interferograms on Estimated ALT

Whereas previous applications of the ReSALT technique have utilized multiple interferograms collected over an individual thaw season, the 2017 ABoVE airborne campaign is limited to a single interferogram collected over each study site, with the first scene collected at the start of the 2017 summer thaw season, and the second scene collected shortly before autumn refreeze the same year. The lack of multiple interferograms over a given thaw season makes retrievals of the active layer thickness sensitive to any errors present in the single interferogram available at each site in the 2017 ABoVE airborne dataset. To demonstrate the sensitivity of single‐interferogram ALT retrievals to interferometric error terms, we simulate an airborne observation scheme with multiple interferograms collected per thaw season and quantify the consequent increase in the precision of ALT estimates.

**Figure 11:**
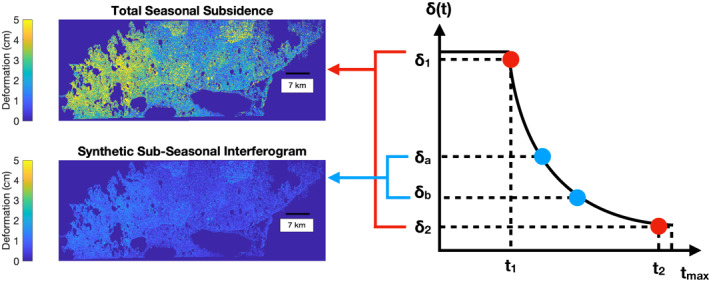
Scaling method used to generate a network of several synthetic airborne interferograms collected over a single thaw season. The total seasonal subsidence over the entire thaw season is estimated from the single real interferogram (red dots; top left image) using the degree day correction from Section 4.4. Synthetic interferograms (blue dots; bottom left image) are generated by rescaling the total seasonal subsidence based on the synthetic acquisition dates and adding thermal and decorrelation noise terms.

To simulate an airborne observation scheme with multiple interferometric observations over a single thaw season, we first estimate the total seasonal subsidence by scaling the single interferometric observation by the ADDT relationship described in Section 4.4. We use gridded DAYMET temperature reanalysis data to calculate the ADDT curve for the 2017 thaw season over each UAVSAR scene (Thornton et al., 2016). Next, we generate a synthetic series of N interferograms. First, we generate a series of day‐of‐year pairs for each synthetic interferogram and generate the corresponding unwrapped interferogram by scaling the original unwrapped interferogram by the ratio of the synthetic differential ADDT value to the original ADDT value (see Figure 11). We then add two noise terms to each synthetic interferogram: a white Gaussian noise term and a decorrelation noise term that is proportional to the Cramer‐Rao bound of phase variance (Michaelides, Zebker, & Zheng, 2019). This second term simulates interferometric noise due to signal decorrelation, while the former term simulates thermal noise in the radar instrument.

Once we have generated a collection of synthetic interferograms, we perform a linear least squares inversion to infer the total seasonal subsidence based on measured deformation of the network of interferograms. The relevant equation is:

(11) $$\left[\begin{array}{c} \Delta \delta_{i}\\ .\\ .\\ .\\ \Delta \delta_{N} \end{array}\right]=\left[\begin{array}{c} \sqrt{ADDT_{2,i}-ADDT_{1,i}}\\ \ldots \\ \ldots \\ \ldots \\ \sqrt{ADDT_{2,N}-ADDT_{1,N}} \end{array}\right]\left[\begin{array}{c} S \end{array}\right]$$

where the [*N* × 1] vector of interferometric deformation Δ*δ* is related to the best‐fitting estimate of the seasonal deformation *S* by an [*N* × 1] matrix of differential ADDT values as described in Section 4.4. Equation 11 can be inverted for every possible collection of synthetic interferograms generated.

**Figure 12:**
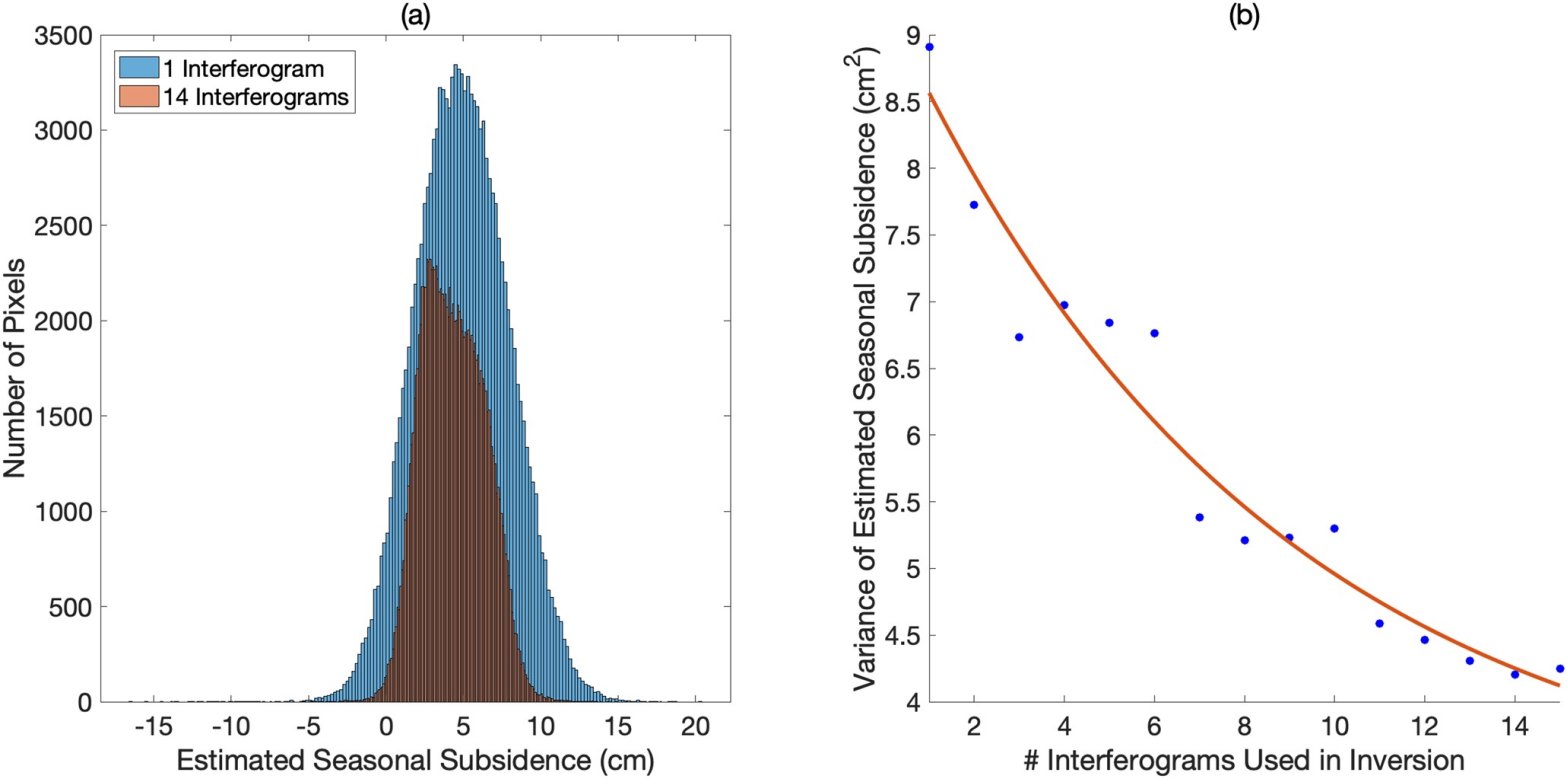
(a) Histogram of best‐fitting seasonal subsidence estimate from ReSALT technique using a single interferogram (blue) and 14 interferograms collected at different times throughout a single thaw season (red). (b) Variance of best‐fitting seasonal subsidence estimate from ReSALT technique as a function of the number of interferograms used in the inverse problem. As the number of interferograms in the inverse problem is increased, the estimate of seasonal subsidence becomes increasingly certain.

Analysis of the simulation described above reveals that increasing the network size of interferograms (the number of interferograms) used during ALT estimation results in a more precise estimate of the seasonal deformation. As the number of interferograms *N* is increased, both the SD and the MAD of the distribution of best‐fitting seasonal subsidence decrease (Figure 12). Put another way, the observation strategy in the ABoVE campaign, which relies on a single observation per thaw season, results in estimates of seasonal subsidence and active layer thickness with larger uncertainties than previous spaceborne InSAR studies which relied on larger interferogram networks.

## 7 Conclusion

Previous InSAR studies of permafrost dynamics have relied on large networks of spaceborne interferometric data. The L‐band and P‐band radar dataset collected during the 2017 ABoVE airborne campaign represents an unprecedented opportunity to study permafrost dynamics with L‐band and P‐band radar at fine spatial resolution. In this study, we have introduced several calibration techniques which we developed to adapt existing interferometric algorithms to the unique collection strategy of the 2017 airborne dataset.

The fine native resolution of the UAVSAR radar compared to spaceborne InSAR instruments, coupled with the high heterogeneity of tundra surfaces and the “single interferogram per season” collection strategy resulted in larger deformation uncertainties compared with comparable spaceborne InSAR studies. This necessitated an uncertainty estimation technique that explicitly relies on the statistics of the measured interferometric phase, as well as a multi‐point calibration technique for referencing relative unwrapped phase. A paucity of reliable meteorological stations over much of the ABoVE domain prevented atmospheric phase error correction techniques which rely on reanalysis data. Further, lack of in situ temperature data resulted in uncertainty in the onset of spring thaw. We developed a high pass spatial filter to remove atmospheric noise from individual UAVSAR interferograms, as well as a degree day calibration that is insensitive to uncertainty in the onset of thaw with which to extrapolate total seasonal subsidence from measured subsidence. Finally, we developed a technique to correct phase unwrapping errors that result from discontinuous phase regions in unwrapped interferograms.

While these techniques were developed explicitly for the 2017 ABoVE airborne campaign, several have general applicability to a range of InSAR‐based studies. Furthermore, several results presented here have direct implications for comparison and integration of airborne and spaceborne InSAR datasets for analysis of permafrost dynamics (Xu et al., 2021, submitted to this special issue). The growth of airborne InSAR datasets will allow for novel applications of InSAR analysis in periglacial environments.

## Figures and Tables

**Figure A1:**
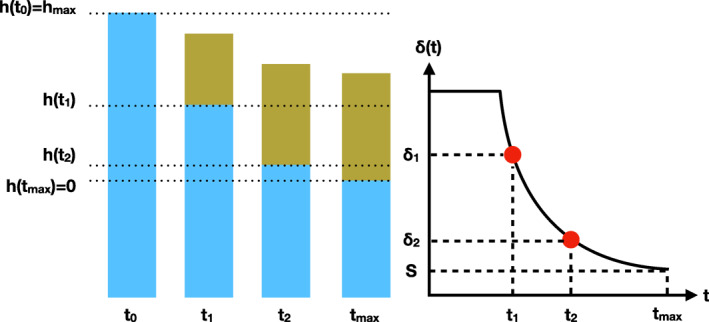
Schematic of the proposed modification to the ADDT‐deformation technique proposed by Liu et al. (2012). Left: At the onset of thaw *t*_0_, the entire soil column is frozen (light blue). Thawing of the permafrost induces a downward migration of the vertical position of the thaw front *h*(*t*) (boundary between unfrozen, brown, active layer and frozen, blue, permafrost). The height of the thaw front is referenced to the minimal position of the thaw front, corresponding to the time at which the active layer has maximally thawed, *t*_*max*_. Right: Deformation of the soil column is plotted as a function of time, and observed at two times, *t*_1_ and *t*_2_, with corresponding deformations of *δ*_1_ and *δ*_2_ (illustrated by red circles). The total seasonal subsidence, *S*, is reached at the end of thaw, before freeze up, and is associated with a corresponding time, *t*_*max*_.

## Data Availability

All UAVSAR data and metadata used in this study is available on https://uavsar.jpl.nasa.gov/cgi-bin/data.pl.

## Appendix A Degree Day Correction

### A.1

Seasonal thawing of the active layer is determined, to first order, by the temperature gradient between the frozen base of the active layer and above‐zero air temperatures (Harlan & Nixon, 1978). As above‐zero degree‐days accumulate over a thaw season, this temperature gradient induces a progressive thawing of the active layer as the 0˚ isotherm gradually moves downward (Brown et al., 2015). In previous studies, where InSAR datasets containing multiple observations per thaw season were available, a simple relationship between subsidence and accumulated degree days of thaw (ADDT) was employed to extrapolate the total seasonal subsidence of the soil column from the measured deformation (Liu et al., 2012; Michaelides, Schaefer, et al., 2019; Schaefer et al., 2015). This approach implicitly assumes that the date at which thaw onsets is known and extrapolates the total seasonal subsidence by referencing the measured deformation values to this assumed date of zero deformation. However, due to the quadratic nature of the relationship between thaw subsidence and ADDT, uncertainty in the onset of thaw can propagate into large uncertainties in the extrapolated seasonal subsidence using this method.

We propose the following modification to the approach first outlined in Liu et al. (2012) to produce estimates of seasonal subsidence that are less sensitive to uncertainty in the onset of thaw date. Starting with Stefan's Law (Poirier & Geiger, 2016), we assume purely vertical heat transport, no warming from the Earth's interior, and a known air temperature at the air‐ground surface boundary. Under these assumptions, the heat flux of thermal energy at the top of the column will be balanced by thawing of the column, and a downward progression of the thaw front *h*(*t*):

(A1) $$Q=\frac{-\kappa_{\rho }}{h\left(T\right)}\left(T_{top}-T_{bot}\right)=\rho_{\rho }L\frac{\delta h}{\delta t}$$

where *Q* is the heat flux at the top of the frozen soil column, *κ*_*rho*_ is the heat conductivity of the frozen soil column, *T*_*bot*_ and *T*_*top*_ are the temperatures at the bottom and top boundaries of the frozen component of the soil column, respectively; *h*(*t*) is the height of the thaw front as a function of time (defined such that *h*(*t* = 0) = *h*_*m*_ *ax* and *h*(*t* → *∞*) → 0 if one neglected freeze up), *ρ*_*ρ*_ is the bulk density of the frozen soil column, and *L* is the latent heat of thawing.

Rearranging Equation A1 as follows:

(A2) $$h\left(T\right)\delta h=\frac{-\kappa_{\rho }}{\rho_{\rho }L}\left(T_{top}-T_{bot}\right)\delta t$$

We can then take the definite integral of Equation A2 between two times of observation, *t*_1_ and *t*_2_. First, let us define $N=\frac{-\kappa_{\rho }}{\rho_{\rho }L}$, as it is independent of time:

(A3) $$\int_{T_{1}}^{T_{2}}h\left(T\right)\delta h=-N\int_{T_{1}}^{T_{2}}\left(T_{top}-T_{bot}\right)\delta t$$

Under the assumption that the base of the frozen soil column remains below freezing, the integral on the right‐hand side of Equation A3 evaluates to a linear difference in the ADDT between the two observation times, and Equation A3 integrates to:

(A4) $$h{\left(t_{2}\right)}^{2}-h{\left(t_{1}\right)}^{2}=-2N\left(ADDT\left(t_{2}\right)-ADDT\left(t_{1}\right)\right)$$

Next we re‐express *h*(*t*_1_) = *h*(*t*_2_) + *δh*, where *δh* is the vertical change in the thaw front between the two observation times, insert this change of variables into Equation A4, expand, and simplify to:

(A5) $$2h\left(t_{2}\right)\delta h+{\left(\delta h\right)}^{2}=2N\left(ADDT\left(t_{2}\right)-ADDT\left(t_{1}\right)\right)$$

From Figure A1 and Equation A5, it is clear that the downward progression of the thaw front proceeds rapidly at the onset of thaw, and gradually retards as the thaw season progresses, consistent with empirical field observations (Hinkel & Nicholas, 1995; Nelson et al., 1997). Toward the end of the thaw season, an incremental increase in the ADDT produces an increasingly negligible progression of the thaw front. If the second observation is taken toward the end of the thaw season, then *h*(*t*_2_) ≈ 0 and Equation A5 simplifies to:

(A6) $${\left(\delta h\right)}^{2}\approx 2N\left(ADDT\left(t_{2}\right)-ADDT\left(t_{1}\right)\right)$$

We can see that a change in the thaw depth is proportional to the square root of the difference in degree days of thaw between the two observation times:

(A7) $$\delta h\propto \sqrt{\left(ADDT\left(t_{2}\right)-ADDT\left(t_{1}\right)\right)}$$

The deformation model derived in Liu et al. (2012) relates a change in the thaw depth to a corresponding subsidence of the soil column:

(A8) $$\Delta \delta =PS_{0}\frac{\rho_{w}-\rho_{i}}{\rho_{i}}\delta h$$

where Δ*δ* is the subsidence of the soil column, *P* and *S*_0_ are the depth‐averaged porosity and saturation of the soil column, respectively (their product is the depth‐averaged water content), and *ρ*_*w*_ and *ρ*_*i*_ are the densities of water and ice, respectively. By inserting Equation A7 into Equation A8, we can see that the subsidence of the soil column observed between times *t*_1_ and *t*_2_ is therefore:

(A9) $$\Delta \delta \propto 2NPS_{0}\frac{\rho_{w}-\rho_{i}}{\rho_{i}}\sqrt{\left(ADDT\left(t_{2}\right)-ADDT\left(t_{1}\right)\right)}$$

and the subsidence of the soil column is directly proportional to the square root of the difference in degree days of thaw between the two observation times.

As each interferogram pair in the 2017 UAVSAR dataset partially spans the 2017 thaw season, we scale the measured deformation in each interferogram based on the relative change in ADDT:

(A10) $$S=\frac{\sqrt{ADDT_{max}}}{\sqrt{ADDT_{2}-ADDT_{1}}}\Delta \delta$$

where *S* is the extrapolated maximum seasonal thaw depth over the entire thaw season, Δ*δ* is the measured subsidence, and *ADDT*_1_, *ADDT*_2_, and *ADDT*_*max*_ are the ADDT for the first scene, second scene, and over the entire thaw season, respectively.

The difference between the degree day correction derived above and the correction derived in Liu et al. (2012) lies in the boundary condition assumptions made to solve Equation A1. The Liu model implicitly assumes *h*(*t* = 0) = *h*_*max*_, while the model proposed here assumes *h*(*t*_2_) ≈ 0. In other words, the Liu model assumes that the time at which the onset of thaw, and therefore surface subsidence, is known exactly, while the proposed model assumes that the second observation occurs toward the end of the thaw season, such that the soil column is not expected to subside significantly after this observation. By observing Figure A1, we can see that the boundary condition of the Liu model occurs when the derivative of the subsidence is largest, that is, when the behavior is most sensitive to perturbations. As such, uncertainty in the onset of thaw can result in large errors. The proposed model, rather, makes its boundary condition when the derivative of the subsidence function is closest to zero, and therefore least sensitive. As a result, this model is less sensitive to uncertainty in the onset of thaw. Mathematically, the chosen boundary conditions of the proposed model result in a lower‐bound estimate of thaw front migration.
